# The C-terminal propeptide of a plant defensin confers cytoprotective and subcellular targeting functions

**DOI:** 10.1186/1471-2229-14-41

**Published:** 2014-02-05

**Authors:** Fung T Lay, Simon Poon, James A McKenna, Angela A Connelly, Barbara L Barbeta, Bruce S McGinness, Jennifer L Fox, Norelle L Daly, David J Craik, Robyn L Heath, Marilyn A Anderson

**Affiliations:** 1 La Trobe Institute for Molecular Science, La Trobe University, Melbourne VIC 3086, Australia; 2 School of Botany, University of Melbourne, Melbourne VIC 3010, Australia; 3 Institute for Molecular Bioscience, University of Queensland, Brisbane QLD 4072, Australia

**Keywords:** Plant defensin, Phytotoxicity, Vacuolar targeting, Transgenic cotton

## Abstract

**Background:**

Plant defensins are small (45–54 amino acids), basic, cysteine-rich proteins that have a major role in innate immunity in plants. Many defensins are potent antifungal molecules and are being evaluated for their potential to create crop plants with sustainable disease resistance. Defensins are produced as precursor molecules which are directed into the secretory pathway and are divided into two classes based on the absence (class I) or presence (class II) of an acidic C-terminal propeptide (CTPP) of about 33 amino acids. The function of this CTPP had not been defined.

**Results:**

By transgenically expressing the class II plant defensin NaD1 with and without its cognate CTPP we have demonstrated that NaD1 is phytotoxic to cotton plants when expressed without its CTPP. Transgenic cotton plants expressing constructs encoding the NaD1 precursor with the CTPP had the same morphology as non-transgenic plants but expression of NaD1 without the CTPP led to plants that were stunted, had crinkled leaves and were less viable. Immunofluorescence microscopy and transient expression of a green fluorescent protein (GFP)-CTPP chimera were used to confirm that the CTPP is sufficient for vacuolar targeting. Finally circular dichroism and NMR spectroscopy were used to show that the CTPP adopts a helical confirmation.

**Conclusions:**

In this report we have described the role of the CTPP on NaD1, a class II defensin from *Nicotiana alata* flowers. The CTPP of NaD1 is sufficient for vacuolar targeting and plays an important role in detoxification of the defensin as it moves through the plant secretory pathway. This work may have important implications for the use of defensins for disease protection in transgenic crops.

## Background

Plant defensins are a family of small (~5 kDa, 45–54 amino acids), basic, cysteine-rich proteins that are widespread in the plant kingdom and have been described in various tissues and species [1]. Many, but not all plant defensins, have potent antifungal activity and thus have generated interest in their application for control of fungal disease in crop plants [2].

Plant defensins are divided into two classes based on precursor proteins predicted from cDNA clones [1,3]. Class I defensin precursors have an endoplasmic reticulum (ER) signal peptide and a defensin domain. Defensins from this class enter the ER where they are folded and subsequently secreted via the default pathway into the extracellular space [4,5]. Many of the well-characterised seed defensins belong to this class [1].

Class II defensins have an additional C-terminal propeptide (CTPP) of about 33 amino acids. Most members of this class are produced by solanaceous species where they are expressed constitutively in floral tissues and fruit, and are induced in leaves during salt-stress (reviewed in Lay and Anderson [1]). However, a small number of defensins with CTPPs have been discovered outside the Solanaceae such as ZmESR-6 (*Zea mays* Embryo Surrounding Region-6) from maize (Poaceae) [6]. The CTPPs from NaD1 (from the flowers of *Nicotiana alata*), PhD1 and PhD2 (from the flowers of *Petunia hybrida*) are not present on the mature, biologically active defensins [7]. This led to our hypothesis that the CTPP could be a vacuolar targeting signal (VTS) since VTSs are removed from vacuolar proteins during transit to, or deposition in the vacuole and unlike the extracellular class I defensins [4,5] the NaD1 defensin is deposited in the vacuole [3].

The CTPPs of solanaceous class II defensins carry an overall negative charge that closely matches and counter-balances the positive charge of the defensin domain [3]. This led us to ask whether the CTPP could have an additional role in protein folding and/or protein detoxification (i.e. preventing phytotoxicity) [3] like the propeptides on plant α-/β-thionins and mammalian defensins that share an analogous charge disparity between their pro- and mature protein domains [7].

In a recent paper [8], we described the production and performance of transgenic cotton plants expressing the natural NaD1 precursor consisting of the mature 47 amino acid defensin domain flanked by an ER signal peptide and the 33 amino acid CTPP. These plants were phenotypically indistinguishable from the non-transgenic parents and exhibited enhanced resistance to both Fusarium and Verticillium wilt over three years of field trials.

In this paper, we investigated the role of the 33 amino acid CTPP of NaD1 by generating transgenic cotton plants expressing NaD1 without the CTPP to determine its effect on vacuolar targeting and potential phytotoxicity. Furthermore, we examined whether the defensin CTPP was sufficient to direct cytosolic green fluorescent protein to the vacuole.

## Results

### Production of transgenic cotton

**Figure 1:**
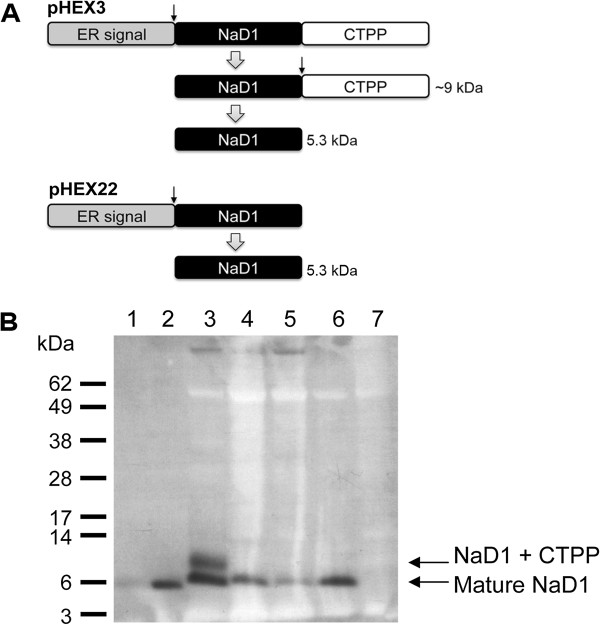
**Production of NaD1 in leaves of transgenic cotton transformed with pHEX22 or pHEX3. (A)** Diagram of the defensin precursors encoded by pHEX3 and pHEX22. Both precursors have ER signal peptides that direct the protein into the secretory pathway. pHEX22 encodes the mature NaD1 defensin of 5.3 kDa and pHEX3 encodes NaD1 with a C-terminal propeptide. This propeptide is removed from the ~9 kDa intermediate during transit to or storage in the vacuole where the mature 5.3 kDa defensin accumulates. Processing sites are indicated by black arrows. **(B)** Immunoblot of leaf tissue using an anti-NaD1 antibody. Lane 1: 50 ng purified NaD1 from *N. alata* flowers, lane 2: 150 ng purified NaD1 from *N. alata* flowers, lane 3: line D1 (homozygous T_3_), lane 4: line 78.131.1 (T_1_), lane 5: line 83.68.1 (T_1_), lane 6: 83.68.1 (T_1_), lane 7: non-transgenic Coker 315. Size markers are SeeBlue Plus2 (Invitrogen).

Twelve PCR-positive primary transgenic plants (T_0_) representing 10 transgenic events were produced with the pHEX22 (SP-NaD1ΔCTPP) construct (Table 1 and Figure 1A). All of these plants had detectable levels of NaD1 in the leaves as determined by ELISA (data not shown). An immunoreactive protein of ~5 kDa protein was observed on protein blots of leaf extracts from lines 78.131.1 and 83.68.1 consistent with the size of mature NaD1 (Figure 1B). In contrast, immunoreactive proteins of ~5 kDa and ~9 kDa were present in leaf extracts from the transgenic cotton line D1 (pHEX3: SP-NaD1-CTPP), consistent with the sizes of mature NaD1 and proNaD1 (NaD1-CTPP), respectively (Figures 1A and B). Fifty-eight primary transgenic plants representing 38 transgenic events were produced with the pHEX3 (SP-NaD1-CTPP) construct. Ten plants (17%), all representing different events, were either infertile and/or had distorted or small leaves. The remaining plants had a normal phenotype and were fertile. In contrast, 10 of the 12 primary pHEX22 transgenic plants (83%), representing nine events, exhibited distorted or small leaves and/or short internodes (Table 1). Furthermore, seven of these 10 primary transformants were infertile (Table 1). Two of the primary pHEX22 transformants (from two events) had a normal phenotype.

**Table 1:** Phenotype of primary transgenic plants transformed with pHEX22 (SP-NaD1ΔCTPP)

**Event**	**Plant no.**	**Phenotype**	**Fertile**
78.131	1	Distorted leaves	Yes
	2	Distorted leaves	Yes
83.23	1	Slightly distorted leaves	No
	2	Normal	Yes
83.54	1	Distorted leaves, short internodes	No
83.67	1	Distorted leaves, short internodes	No
83.68	1	Normal	Yes
83.96	1	Distorted leaves, short internodes	No
83.102	1	Small leaves	Yes
83.111	1	Normal leaves, short internodes	No
83.166	1	Distorted leaves, short internodes	No
83.182	1	Distorted leaves, spindly habit	No

### Characterisation of segregating T_1_ transgenic plants

To determine if the abnormal phenotype of the pHEX22 transformed cotton plants segregated with the *NaD1* gene, seed from the four fertile transgenic events (lines 78.131.1, 83.23.2, 83.68.1 and 83.102.1) were planted in the greenhouse and the germinated T_1_ plants were assessed for NaD1 accumulation, number of transgene inserts and phenotype. PCR screening of the T_1_ plants indicated that the primary transgenic plants (T_0_) of lines 78.131.1 and 83.102.1 had only one copy of the transgene *NaD1* (Table 2). Real-time PCR revealed the distribution of homozygous, hemizygous and null plants expected for a single insertion (Table 2) and Southern blot analysis confirmed single inserts (data not shown). There were very few null plants in the segregating T_1_ populations of lines 83.68.1 and 83.23.2 which is consistent with the presence of more than one transgene insertion in the primary transgenic plants (Table 2).

**Table 2:** **Segregation of T**_**1 **_**plants transformed with pHEX22 (SP-NaD1ΔCTPP)**

**Line**	**No. plants**	**PCR -ve**	**PCR + ve**	**Homozygous**	**Hemizygous**
78.131.1	45	8 (18%)	37 (82%)	14 (31%)	23 (51%)
83.102.1	36	10 (28%)	26 (72%)	8 (22%)	18 (50%)
83.68.1	29	2 (7%)	27 (93%)	MC	MC
83.23.2	21	1 (5%)	20 (95%)	MC	MC

The presence of *NaD1* was determined by PCR. Lines 73.131 and 83.102 plants were identified as homozygous or hemizygous by real time PCR. Values in parentheses indicate the percentage of the tested plants. Lines 83.68.1 and 83.23.2 had more than one DNA insertion so zygosity was not determined. MC = multicopy lines.

Homozygous plants of line 78.131.1 had the highest levels of NaD1 of the pHEX22 transgenics with an average of 6.8 ppm in their leaves (Table 3). Hemizygous plants of this line had about half the NaD1 levels at about 2.6 ppm (Table 3). Line 83.102.1 had less NaD1 than line 78.131.1, with an average of 1.7 ppm and 0.9 ppm in the leaves of homozygous and hemizygous plants, respectively (Table 3). Lines 83.68.1 and 83.23.2 had lower levels of NaD1 at about 1.1 ppm and 0.6 ppm, respectively (Table 3). In comparison, the three homozygous line D1 plants (pHEX3: SP-NaD1-CTPP) that were sown at the same time as the pHEX22 events had about 3.5 ppm NaD1 in their leaves.

**Table 3:** **NaD1 levels and phenotype of segregating pHEX22 T**_**1 **_**plants expressing SP-NaD1**Δ**CTPP and homozygous Line D1 plants expressing SP-NaD1-CTPP**

**Line**	Zygosity*	**Plant no.**	**NaD1 ppm**	**Leaf distortion**	**Fertile**	**Bolls**	**Seed**	**Germination %**
78.131.1	Hom	10	5.0	Severe	Yes	1	11	55
		23	5.9	Severe	No	0		
		34	7.9	Severe	No	0		
		42	8.3	Severe	Yes	1	7	57
		**Ave.**	**6.8**					
78.131.1	Hem	48	2.7	Moderate	Yes	2	20	70
		12	3.0	Moderate	Yes	1	17	100
		44	2.4	Moderate	Yes	2	38	80
		47	2.2	Moderate	Yes	2	16	80
		**Ave.**	**2.6**					
83.102.1	Hom	7	1.5	Mild	Yes	2	25	90
		20	2.0	Mild	Yes	2	40	80
		21	0.6	Mild	Yes	4	39	100
		27	2.6	Mild	Yes	3	33	100
		**Ave.**	**1.7**					
83.102.1	Hem	15	0.8	Slight	Yes	4	31	90
		16	0.7	Slight	Yes	4	43	80
		25	0.9	Slight	Yes	2	49	100
		29	1.0	Slight	Yes	3	32	80
		**Ave**	**0.9**					
83.68.1	MC	1	0.6	None	Yes			
		2	0.7	None	Yes			
		3	1.1	None	Yes			
		4	1.0	None	Yes			
		5	1.0	None	Yes			
		6	2.1	None	Yes			
		**Ave**	**1.1**					
83.23.2	MC	2	0.2	None	Yes			
		3	0	None	Yes			
		4	3.0	None	Yes			
		5	0	None	Yes			
		6	0.1	None	Yes			
		7	0	None	Yes			
		**Ave**	**0.6**					
D1	Hom	1	2.7	None	Yes			
		2	3.6	None	Yes			
		3	4.1	None	Yes			
		**Ave**	**3.5**					

*Hom = homozygous, *Hem* = hemizygous, *MC* = multicopy lines, zygosity not determined. NaD1 levels were determined 6 weeks after sowing. ppm = ng NaD1/mg wet weight tissue.

**Figure 2:**
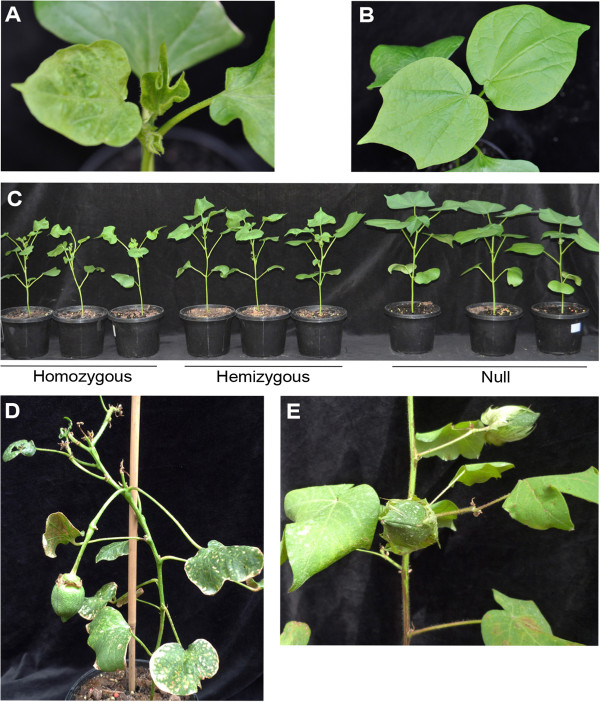
**T**_**1 **_**segregating plants of line 78.131.1. (A)** Close-up of a homozygous plant with distorted leaves, 26 days after sowing. **(B)** Close-up of a null plant with normal phenotype, 26 days after sowing. **(C)** Plants at 41 days after sowing. **(D)** Close-up of mature a homozygous plant, 119 days after sowing. The plant produced one small boll while most flowers and bolls aborted. **(E)** Close-up of bolls on a mature null plant, 119 days after sowing.

Phytotoxicity was observed in the pHEX22 (SP-NaD1ΔCTPP) segregating T_1_ plants that was correlated with the level of NaD1 in the leaves. All the leaves on line 78.131.1 homozygous plants, which had the highest levels of NaD1, were severely distorted (Table 3, Figures 2A and C) and these plants were shorter than the null plants. Furthermore, line 78.131.1 homozygous plants were either infertile or produced fewer bolls that were smaller and had less seed than the null plants (Table 3, Figure 2D). All null plants had a normal phenotype (Figures 2B and E) whereas the hemizygous 78.131.1 plants had a phenotype that was intermediate between that of the homozygous and null plants. That is, there was moderate distortion of the leaves (Table 3, Figure 2C) and some reduction in seed production. In comparison, line D1 homozygous plants (pHEX3: SP-NaD1-CTPP) had a normal phenotype (Table 3).

**Figure 3:**
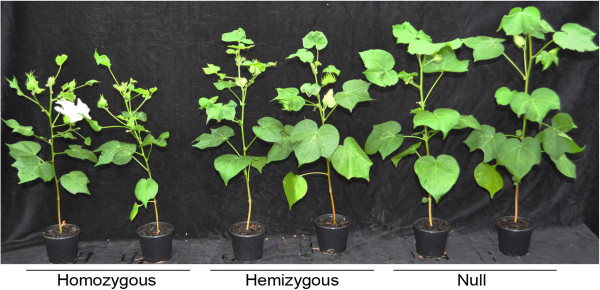
**T**_**1 **_**segregating plants of line 83.102.1.** Representative homozygous, hemizygous and null plants from line 83.102.1 87 days after sowing.

Homozygous plants of line 83.102.1 exhibited some phytotoxicity but it was much less obvious than for line 78.131.1 and was correlated with lower NaD1 expression levels in leaf tissue. There was no difference in leaf morphology between line 83.102.1 transgenic plants and the nulls in the first few weeks, but as the plants matured, some leaf distortion was observed (Table 3). At 87 days after sowing, when plants were flowering, the homozygous plants were shorter and more spindly than the null plants (Figure 3). Hemizygous line 83.102.1 plants had an intermediate phenotype between homozygous and null plants (Figure 3). All line 83.102.1 transgenic plants were fertile although some bolls were smaller than the bolls from null plants (Table 3). The T_1_ line 83.68.1 and 83.23.2 plants had, on average, less NaD1 at 1.1 and 0.6 ppm than the other two transgenic lines, respectively. They had normal phenotype and were fertile (Table 3).

### Cellular structure of leaves from transgenic cotton plants

**Figure 4:**
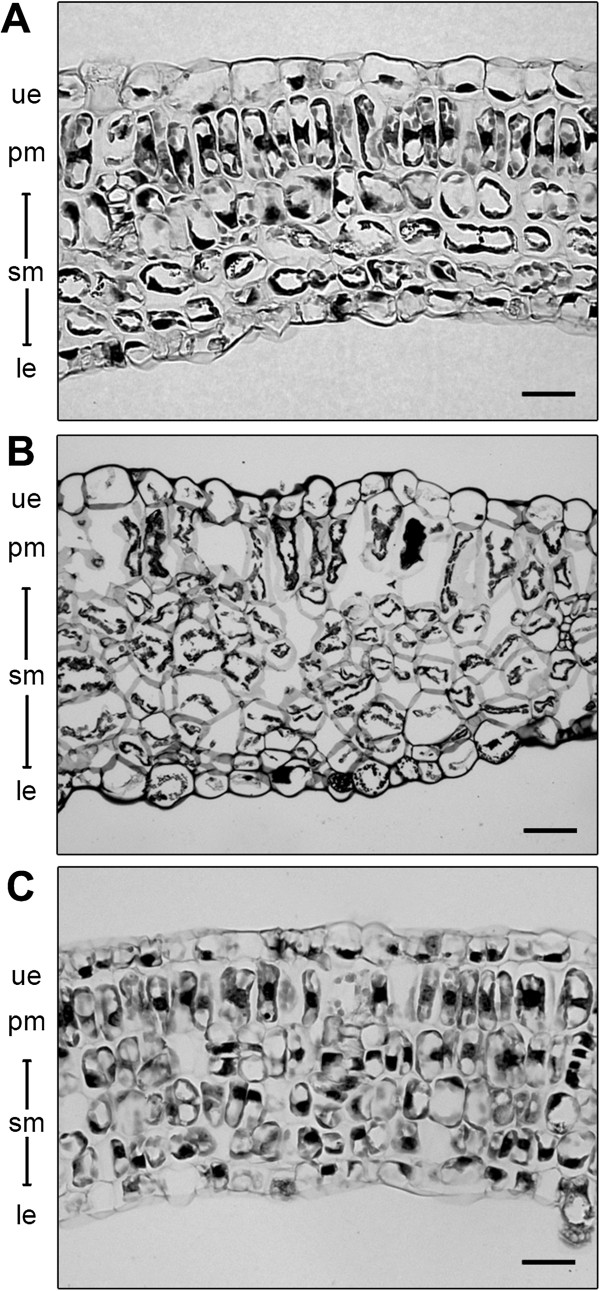
**Cellular organisation of transgenic cotton plants expressing NaD1 from pHEX3 and pHEX22.** Longitudinal sections of leaves from **(A)** line D1, **(B)** line 78.131.1 and **(C)** non-transgenic control plants (ue, upper epidermis; pm, palisade mesophyll; sm, spongy mesophyll; le, lower epidermis). Scale bars = 50 μm.

Leaves from the non-transgenic Coker control and line D1 (transformed with pHEX3) plants presented characteristic, well-ordered arrangements of upper epidermal, palisade mesophyll, spongy mesophyll and lower epidermal cells (Figure 4). In contrast, cells in the leaves of homozygous line 78.131.1 plants were more irregular in shape and the cell layers were disordered. This observation may explain the altered ('wrinkly’) leaf morphology presented in Figures 2 and 3.

### Subcellular location of NaD1 in transgenic cotton plants

**Figure 5:**
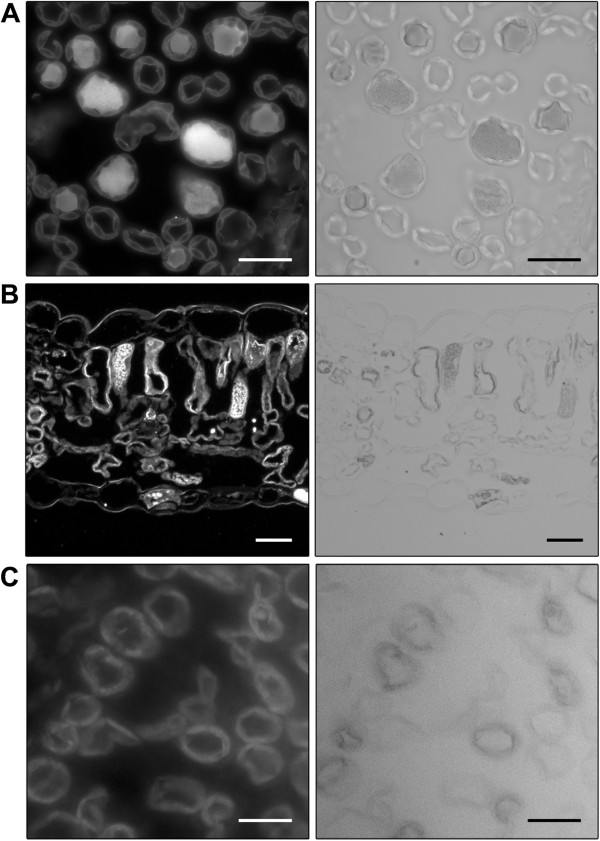
**Subcellular location of NaD1 in stably transformed cotton plants expressing NaD1 from pHEX3 and pHEX22.** Leaf sections from **(A)** line D1, **(B)** line 78.131.1 and **(C)** non-transgenic control plants used for immunofluorescence with anti-NaD1 antibodies (left panels). The right panels are differential interference contrast images of the cells shown in the left panels. All sections are longitudinal sections. Scale bars = 50 μm.

Sections from leaves taken from equivalent positions on the homozygous line D1 (pHEX3) and line 78.131.1 (pHEX22) transgenic plants and non-transgenic Coker control plants were probed with anti-NaD1 antibodies and Alex Fluor 568^®^-labelled secondary antibodies to examine the subcellular location of the expressed NaD1. NaD1 was present in the vacuoles of mesophyll cells from line D1 (Figure 5A) but was confined to the periphery of cells in sections from line 78.131.1 (Figure 5B). The antibody did not bind to the leaf sections from the non-transgenic control plants (Figure 5C).

### Examination of subcellular targeting of the GFP chimeras

**Figure 6:**
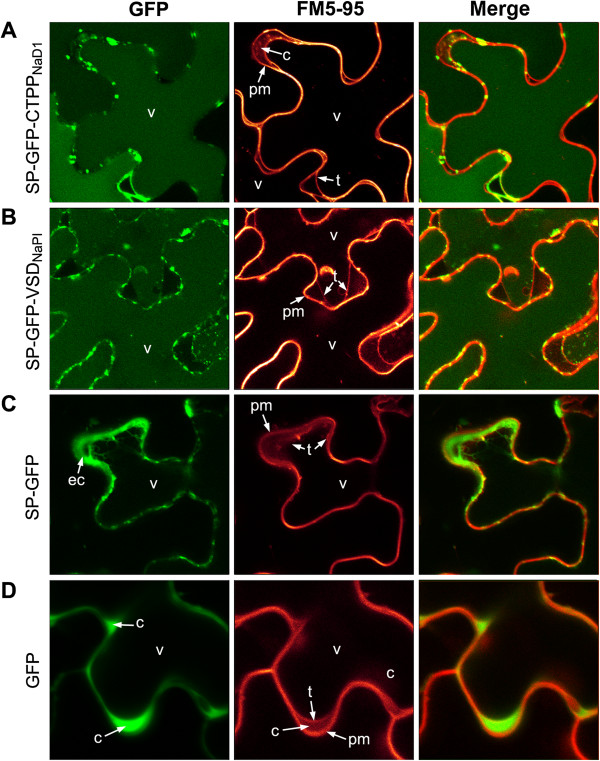
**Location of GFP in *N. benthamiana *cells after transient expression of various GFP-chimera constructs.** Micrographs showing the location of GFP produced during transient expression of **(A)** SP-GFP-CTPP_NaD1_, **(B)** SP-GFP-VSD_NaPI_, **(C)** SP-GFP or **(D)** GFP constructs in the leaves of *N. benthamiana*. The left panels show GFP fluorescence, the middle panels reveal fluorescence from the FM5-95 dye and the right panels are merge images (c, cytoplasm; ec, extracellular; pm, plasma membrane; t, tonoplast; v, vacuole).

In order to assess whether the CTPP from NaD1 was sufficient to direct an otherwise cytosolic protein to the plant vacuole, constructs encoding green fluorescent protein (GFP) linked to the CTPP from NaD1 or the known C-terminal vacuolar sorting determinant (VSD) of NaPI were expressed transiently in *N. benthamiana* leaves. Micrographs showing the location of the GFP (left panels) produced during transient expression of the various constructs in *N. benthamiana* leaves are presented in Figure 6. The middle panels are equivalent sections showing membranes highlighted by the FM5-95 dye while the right panels are an overlay of the two images. Spectral analysis of emitted fluorescence was used to confirm the specificity of the signals for GFP and the FM5-95 dye [9,10].

GFP was located in the vacuole of cells expressing SP-GFP-CTPP_NaD1_ (Figure 6A). In these cells, the junction between the vacuole and the cytoplasm was highlighted by FM5-95 dye which stained both the plasma membrane and the tonoplast. A similar result was obtained with the positive control SP-GFP-VSD_NaPI_ construct (Figure 6B). In cells expressing the GFP construct with an ER signal peptide (SP-GFP) but no CTPP or VSD, GFP was located in the extracellular space, with fluorescence concentrated around the periphery of the cell (Figure 6C). This contrasted with the GFP construct lacking both the ER signal (SP) and CTPP/VSD sequences where the expressed GFP was present in the cytoplasm, which was outlined by the fluorescently labelled plasma and tonoplast membranes (Figure 6D).

### Structure of the NaD1 CTPP

**Figure 7:**
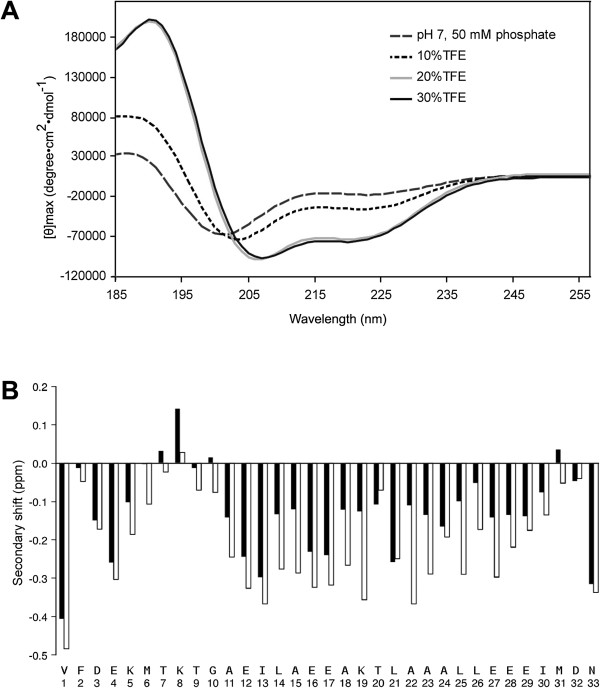
**Structural analysis of the NaD1 CTPP. (A)** CD spectrum of the 33 amino acid CTPP of NaD1 in 50 mM phosphate buffer pH 7.0 (dashes), 10% TFE (dots), 20% TFE (grey) and 30% TFE (black). **(B)** Secondary shift analysis of the CTPP in aqueous solution (black bars) and in 20% TFE (white bars). The secondary shifts were calculated by subtracting random coil Hα shifts [12] from the Hα shifts.

Circular dichroism (CD) and NMR spectroscopy were conducted to determine whether a 33 amino acid synthetic peptide corresponding to the CTPP from NaD1 adopts a helical structure as reported for several other C-terminal vacuolar sorting determinants (VSDs) [11]. The CD spectra indicated a propensity for helical structure that was stabilised in the presence of trifluoroethanol (TFE), as shown in Figure 7A. The NMR data were consistent with this interpretation and analysis of the secondary shifts, shown in Figure 7B, indicated that residues 10–29 form a helix in solution. The secondary shifts for these residues became more negative on addition of TFE, again indicating that the helical structure was stabilised in the presence of TFE (Figure 7B).

## Discussion

We have discovered that the class II defensin, NaD1, can be phytotoxic in transgenic cotton plants when expressed without its 33 amino acid CTPP. This phytotoxicity was manifested in the leaves of transgenic plants as distortions in the cellular architecture that presented as a wrinkly phenotype at the macroscopic level. The plants were stunted and had short internodes, but more critically, were often infertile or produced small bolls with reduced seed number. The phytotoxicity was most obvious in plants that had accumulated about 7 ppm NaD1 in their leaves and was not detectable at NaD1 levels of about 1 ppm or less. The phenotype segregated with *NaD1* in the T_1_ generation, demonstrating that it had been caused by expression of *NaD1* and was not an artefact that had been introduced during tissue culture. This is supported by the observation that 10 out of the 12 primary transformants produced with the SP-NaD1ΔCTPP construct had an abnormal phenotype. This is distinguished from the low number of plants (less than 20%) with an unusual phenotype that normally arise from the cotton embryogenesis regeneration method used to produce the transgenic cotton (unpublished data). This effect was most likely responsible for the 10 plants out of 58 primary transformants (17%) with the SP-NaD1-CTPP construct that were either infertile and/or exhibited distorted or small leaves. Thus, phytotoxicity was not evident in plants when NaD1 was expressed with its cognate CTPP.

Line D1 plants have been assessed in greenhouse bioassays for resistance to *Fusarium oxysporum* f. sp. *vasinfectum* (Fov) and subsequently in field trials over three growing seasons in fields naturally infested with Fov and in two growing seasons in fields naturally infested with *V. dahliae*. In both Fov or *V. dahliae* infested soils, line D1 plants had higher survival rates, greater tolerance to the pathogens and 2- to 4-fold increases in lint yield compared to the non-transgenic control plants [8]. Line D1 plants typically accumulated about 3–6 ppm NaD1 in their older leaves and did not exhibit any detrimental agronomic features such as short internodes, decreased height or fertility relative to the non-transgenic parent. Additionally, when the plants were grown in non-diseased soil there was no yield penalty or difference in lint quality when compared to the non-transgenic control plants [8].

Almost 20 unique plant defensins have been transformed into more than 10 different plant species ranging from Arabidopsis to monocot and dicot crop species in an attempt to enhance resistance to a variety of fungal diseases [13]. Given the number of plant defensins tested, the number of crop species transformed and reports of enhanced disease resistance, it is interesting that none have been developed into a commercial trait. One of the most promising reports was from Gao and co-workers [4] who demonstrated that constitutive expression of the class I defensin alfAFP in potatoes conferred enhanced resistance to *Verticillium dahliae.* This enhanced resistance was maintained in the laboratory and under field conditions over several years at different geographical sites. Much later they reported that plants expressing alfAFP had smaller tubers [14].

To date, the majority of these studies have used class I defensins. In reports where it was explicitly stated, the plants had no obvious deleterious effects from defensin expression. However yield was not reported in any of the glasshouse or field trials and thus, the effects of constitutive expression of class I plant defensins have not been fully evaluated. As reported here, removal of the CTPP from the class II defensin NaD1 to make it more like a class I defensin resulted in abnormal morphology and loss of fecundity. Likewise, Ghag and co-workers have recently demonstrated that the class II petunia defensins [15] are not toxic when expressed in bananas but become phytotoxic when expressed without their cognate CTPPs [16]. Transformation experiments using these defensins without their CTPP produced significantly fewer embryos and these did not develop into mature transgenic banana plants [16].

It is interesting to contemplate why class I defensins are not phytotoxic to their own host plants when they are naturally expressed without a CTPP. Either they do not accumulate to the same high levels as the class II defensins or they have a different mechanism of action. Examination of the three-dimensional structures of the mature domains of various defensins does not indicate any obvious differences between those of class I or class II defensins [1,7,17]. They all adopt comparable and superimposable folds centred on the cysteine-stabilized αβ motif comprising an α-helix and a triple stranded antiparallel β-sheet that is stabilized by a network of four disulfide bonds [7,17,18].

However, although the three-dimensional structure is conserved, the amino acid sequence of plant defensins is highly variable apart from the eight highly conserved cysteine residues, serine 8, an aromatic residue at position 11, glycine 13 and glutamates at position 29 and 34 (numbering relative to RsAFP2) [1]. This has led to the hypothesis that defensins have a conserved scaffold that presents surface loops that are highly variable in sequence and that this variability accounts for the diverse range of biological activities that have been ascribed to defensins [1,19]. NaD1 and other class II defensins from the Solanaceae are thus likely to have a different mechanism of antifungal activity to antifungal class I defensins [13,19]. NaD1, for example, interacts with a specific target on the fungal cell wall before it accesses and permeabilises the plasma membrane and enters the cytoplasm where it may interact with a specific target in the cytoplasm and initiate cell death [20,21]. Psd1, the class I defensin from peas, also enters the fungal cell and travels to the nucleus where it inhibits cell cycle [22]. In contrast, the class I defensins from radish (RsAFP2), dahlia (DmAMP1) and alfalfa (MsDef1) do not enter the cell but interact with specific sphingolipids in the plasma membrane and cell wall leading to membrane disruption and cell death [23-26]. In another mechanism, plant defensins such as MsDef1 and ZmES4 have been reported to interact with ion channels [27,28].

We have previously suggested that the difference in net charge between the mature defensin domain and the CTPP of class II defensins may have evolved as a strategy to prevent deleterious effects on the host cell by making the defensin less reactive (possibly mediated via electrostatic and hydrophobic interactions) [1,3]. This could reduce the likelihood of defensin aggregation or undesirable interactions with other proteins and membranes in the cell during folding and transport through the endomembrane system. In effect, the CTPP could act as an intramolecular chaperone and impart transport competency to the prodefensin.

It is likely that part of the cytoprotective function that is mediated by the CTPP in NaD1 (and probably other class II defensins) is inextricably linked to its vacuolar targeting function. In this paper, we showed by two approaches that the CTPP of NaD1 is necessary and sufficient for vacuolar targeting. Immunofluorescence microscopy on the stably transformed cotton plants revealed a different location for NaD1 expressed with or without its CTPP. When the CTPP was present, NaD1 was targeted to the vacuole and when it was absent, NaD1 was directed to the periphery of the cell. This is consistent with the observations for the well-characterised class I defensins such as RsAFP2 from radish and MsDef1 from alfalfa, which have an extracellular location [4,5]. The observation that the NaD1 CTPP can direct cytosolic GFP to the vacuoles of *N. benthamiana* plants in transient expression studies demonstrates its sufficiency as a vacuolar sorting determinant.

**Figure 8:**
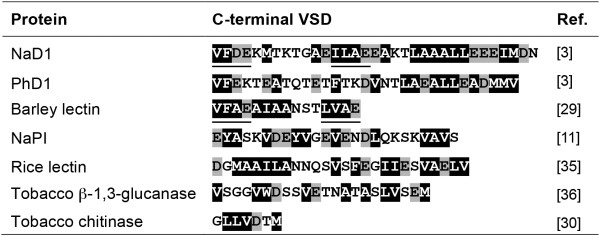
**Comparison of the CTPP of NaD1 and PhD1 with C-terminal vacuolar sorting determinants from other plant proteins.** The acidic amino acids are boxed in grey and the hydrophobic amino acids are boxed in black. NaD1 and barley lectin have two related tetrapeptide motifs that are underlined.

The sequence of the CTPPs from NaD1 and other class II plant defensins (such as PhD1) were compared to other well characterised vacuolar sorting determinants (VSDs) from other plant proteins (Figure 8). The defensin CTPPs are longer, that is, about 33 amino acids in length compared to 15 amino acids for the C-terminal VSD of barley lectin [29] or seven for tobacco chitinase [30]. Furthermore, while no consensus motif has been identified for C-terminal VSDs, they are all enriched in hydrophobic and acidic amino acids (Figure 8). The first half of the NaD1 CTPP also shares two tetrapeptide motifs with the barley lectin VSD; VFDE and ILAE in the NaD1 CTPP and the motifs VFAE and LVAE in the barley lectin VSD (underlined in Figure 8). Others have reported that as few as three or four amino acids can function as vacuolar sorting signals in plants [31-34], including the VFAE or LVAE motifs of barley lectin [31]. The defensin CTPPs may be longer than normal vacuolar sorting motifs because of their dual function in targeting and detoxification.

We demonstrated that the NaD1 CTPP forms a helical structure in solution like other C-terminal VSDs [11]. From a structural context, it would be interesting to examine the presentation of the CTPP with respect to the mature NaD1 defensin domain. Such information could provide details of possible contacts that could be mediated between the two domains and may form the molecular basis for the protein folding and/or detoxification hypothesis. Alternatively, such information could provide clues as to whether the CTPP is indeed helical in the context of proNaD1 and whether it’s helical structure is important for its vacuolar targeting function as has been proposed for the NaPI C-terminal VSD [11]. Nielsen et al. [11] suggested that the α-helical structure of the NaPI VSD may be a necessary requirement for the sorting signal to be relatively exposed to interact with the sorting receptor. Indeed, when the slightly curved amphipathic α-helical structure of the NaPI VSD was combined by molecular modelling with the structure of one of the adjoining proteinase inhibitor (PI) domains, the VSD seems to protrude from an otherwise compact PI domain [11].

## Conclusions

In conclusion, the class II defensin NaD1 is phytotoxic in transgenic cotton plants when expressed without its CTPP. These plants had distortions in their cellular architecture that presented as a wrinkly leaf phenotype, were typically stunted and often infertile or produced small bolls with reduced seed number. We demonstrated by two approaches that the CTPP of NaD1 is necessary and sufficient for vacuolar targeting and when the CTPP was absent, NaD1 was directed to the periphery of the cell. Thus the CTPP plays an important role in targeting and detoxification of the defensin as it moves through the plant secretory pathway. The CTPP is crucial for the transgenic expression of class II defensins to avoid a yield penalty from phytotoxic side-effects.

## Methods

### Binary vector constructs

The construct encoding SP-NaD1ΔCTPP (ER signal peptide and mature defensin domain without the 33 amino acid CTPP) was generated by PCR using the pHEX3 plasmid DNA [8] as a template with the forward oligonucleotide primer DEF1 (5′-CC*G GAT TC*A TGG CTC GCT CCT TGT G-3′) and reverse oligonucleotide primer DEF3 (5′-GCG *GTC GAC* TTA ACA TGG CTT AGT ACA TAG G-3′). Primers DEF1 and DEF3 introduced *Bam*HI and *Sal*I sites (underlined), respectively. The PCR product was subcloned into the pCR2.1-TOPO vector (Invitrogen, Carlsbad, CA), excised with *Bam*HI and *Sal*I, and cloned between the CaMV 35S promoter and terminator sequences of the pAM9 vector [37]. The resultant expression cassette was excised with *Eco*RI and ligated into pBIN19 binary vector [38]. This construct was named pHEX22. The preparation of pHEX3 is described in [8]. pHEX3 has DNA encoding the entire sequence of the NaD1 precursor (ER signal peptide, defensin domain and CTPP) (GenBank accession no. AF509566).

### Production of transgenic cotton

The pHEX22 construct was transferred to *Agrobacterium tumefaciens* strain LBA4404 and used to infect hypocotyl sections of *Gossypium hirsutum* L. cv. Coker 315 cotton plants. Embryogenic callus was selected on kanamycin at 35 mg/L and embryos were germinated and plantlets transferred to soil as described in [8]. Plantlets were screened by PCR using primers for the CaMV 35S promoter (forward: 5′-CCT TCC TGT ATA TAA GGA AGT-3′, reverse: 5′-GAT AGA TTT GTA GAG AGA GAC-3′). NaD1 protein levels were determined by ELISA using polyclonal anti-NaD1 antibodies [21] as described in Gaspar et al. [8]. Immunoblot analysis was conducted using polyclonal anti-6H.proNaD1 antibodies [3] as described in Gaspar et al. [8]. The transgene copy number was determined by quantitative real-time PCR as described in Yi et al. [39]. Transgenic cotton plants expressing pHEX3 (including line D1) were prepared and tested as described in Gaspar et al. [8].

### Subcellular location of NaD1 in transgenic plants

Leaf segments from non-transgenic, line D1 (transformed with pHEX3) and line 78.131.1 (transformed with pHEX22) plants were fixed in 4% (v/v) paraformaldehyde before paraffin embedding and sectioning. The sections were probed with antibodies raised to hexahistidine-tagged proNaD1 (anti-6H.proNaD1; [3]) (50 μg IgG/mL in blocking solution; 0.2% (v/v) Triton X-100, 1 mg/mL BSA in PBS) for 60 min. The slides were then washed three times with PBS before application of Alex Fluor^®^-labelled secondary antibodies (Molecular Probes, Carlsbad, CA), diluted 1:200 in blocking solution. The sections were visualised on an Olympus BX50 microscope and images were captured using a Spot monochrome camera with Spot RT software (version 3.4, Diagnostic Instruments, Sterling Heights, MI). At the time of fixation, samples were taken from the same leaves for ELISA determination of NaD1 levels as outlined above.

### Generation of GFP-CTPP chimera constructs

Specific oligonucleotide primers and PCR was used to generate a number of green fluorescent protein (GFP) chimera expression cassettes that encoded soluble modified GFP (GenBank accession no. U70495, [40]) in combination with DNA encoding the ER signal peptide (SP) from NaPI (GenBank accession no. U08219, nucleotides 10-97) and the CTPP of NaD1 or the C-terminal vacuolar sorting determinant (VSD) sequence of NaPI (GenBank accession no. U08219, nucleotides 1126-1203). These constructs were referred to as SP-GFP-CTPP_NaD1_ and SP-GFP-VSD_NaPI_, respectively. Two control constructs, SP-GFP and GFP alone, were also made. The PCR amplified products, which incorporated *Bam*HI and *Sal*I sites, were subcloned into the pCR2.1-TOPO vector before they were cloned between the CaMV 35S promoter and terminator sequences of the pAM9 vector and ligated into the pBIN19 binary vector as described for pHEX22.

### Transient expression of GFP chimeras in *Nicotiana benthamiana* leaves and subcellular location of the expressed GFP

Reporter GFP constructs in *Agrobacterium* were infiltrated into leaves and production and subcellular location of the expressed GFP was monitored by confocal microscopy as described in Conlan et al. [41].

### Subcellular location of GFP produced from GFP chimeras

Leaf peels from infiltrated leaf sections were mounted in water on glass slides and glass coverslips were placed on top and secured with melted wax. Samples were examined under a Leica TCS SP2 confocal microscope with a HCX APO 63× W Corr/0.17 CS objective. GFP and FM5-95 fluorescence were monitored at 509 nm and 573 nm, respectively. Ten fields of view were scanned from each leaf section and were captured with confocal LCS 3D software (Leica, Wetzlar, Germany) and Image J (developed by Wayne Rasband, National Institutes of Health, Bethesda, MD; http://rsb.info.nih.gov/ij). Images are representative of at least three independent experiments.

### Peptide synthesis

Boc-L-amino acids were obtained from Merck, 2-(1*H*-Benzotriazol-1-yl)-1,1,3,3-tetramethyluronium hexafluorophosphate (HBTU) was obtained from Richelieu Biotechnologies (Quebec, Canada). Trifluoroacetic acid (TFA), *N*,*N*-diisopropylethylamine (DIEA), and *N*, *N*-dimethylformamide (DMF), all of peptide synthesis grade, were purchased from Auspep (Melbourne, Australia).

The 33 amino acid peptide corresponding to the NaD1 CTPP was assembled using manual solid phase peptide synthesis with Boc chemistry on a 0.5 mmole scale. PAM resin (Applied Biosystems, Foster City, CA) was used and amino acids were added to the resin using HBTU with *in situ* neutralisation [42]. Cleavage of the peptide from the resin was achieved using hydrogen fluoride (HF) with cresol and thiocresol as scavengers (HF:cresol:thiocresol; 9:1:1 v/v). The reaction was allowed to proceed at -5–0°C for 1 h. Following cleavage, the peptide was dissolved in 50% acetonitrile/0.1% TFA and lyophilised. The crude peptide was purified using preparative reverse-phase HPLC (RP-HPLC) on a Vydac C18 column. Gradients of 0.1% aqueous TFA and 90% acetonitrile/0.09% TFA were employed with a flow rate of 8 mL/min and the eluant monitored at 230 nm. Mass analysis was performed on a Sciex (Thornhill, Ontario) triple quadrupole mass spectrometer using electrospray sample ionisation.

### Circular dichroism spectroscopy

Circular dichroism (CD) spectra were measured on a JASCO J810 spectropolarimeter, installed with a standard analysis program. The temperature was maintained at 25°C. Spectra were recorded with a quartz cell of 0.1-cm path length, using an acquisition time of 2 s/nm, with a 1-nm spectral bandwidth, over the wavelength range from 185 to 260 nm. The samples were prepared by dissolving lyophilised peptide in 50 mM sodium phosphate with the pH adjusted to 7.0. Data are represented in molar ellipticities ([θ]_max_, degree · cm^2^ · dmol^-1^). The CD spectra represent an average of at least four accumulations and were corrected by subtracting the buffer base line.

### Nuclear magnetic resonance spectroscopy

Samples for nuclear magnetic resonance (NMR) analysis consisted of ~1 mM peptide in 50 mM sodium phosphate, 90% H_2_O/10% ^2^H_2_O at pH 5.5 or 7.0. TOCSY, NOESY and DQF-COSY spectra were acquired on either a Bruker 600 MHz or AVANCE 750 MHz spectrometer. Spectra were acquired at 283 and 298 K. TOCSY spectra were acquired with an 80 ms mixing time and NOESY spectra with 150 or 250 ms mixing times. Spectra were acquired with 4096 data points in *F*2 and 512 in *F*1, and multiplied with squared sine bell window functions shifted by 90°.

## Abbreviations

NaD1: *Nicotiana alata* defensin 1; CTPP: C-terminal propeptide; VSD: Vacuolar sorting determinant; ELISA: Enzyme-linked immunosorbent assay; CD: Circular dichroism; NMR: Nuclear magnetic resonance; SP: Signal peptide.

## Competing interests

Hexima Limited funded production of the transgenic cotton plants and hold a patent regarding targeting of plant defensins. All the other work was funded by the ARC. Hexima Limited will not in any way gain or lose financially from the publication of this manuscript. Patent WO/2008/128289, Anderson, MA, Heath RL, Lay FT, Poon, S. Modified plant defensins.

## Authors’ contributions

FTL, SP, JAM, RLH and MAA participated in study design, coordination and drafting the manuscript, SP designed and built constructs, AAC and JLF carried out plant molecular analysis, BLB performed microscopy, NLD and DJC conducted the CD and NMR and BSM grew and conducted the plant segregation and phenotypic analysis. All authors read and approved the final manuscript.

## References

[B1] Lay FT Anderson MA Defensins – components of the innate immune system in plants Curr Prot Pep Sci 2005 6 85 101 10.2174/1389203053027575 15638771

[B2] Kaur J Sagaram US Shah D Can plant defensins be used to engineer durable commercially useful fungal resistance in crop plants? Fungal Biol 2011 25 128 135 10.1016/j.fbr.2011.07.004

[B3] Lay FT Brugliera F Anderson MA Isolation and properties of floral defensins from ornamental tobacco and petunia Plant Physiol 2003 131 1283 1293 10.1104/pp.102.016626 12644678 PMC166888

[B4] Gao AG Hakimi SM Mittanck CA Wu Y Woerner BM Stark DM Shah DM Liang JH Rommens CMT Fungal pathogen protection in potato by expression of a plant defensin peptide Nat Biotechnol 2000 18 1307 1310 10.1038/82436 11101813

[B5] Terras FRG Eggermont K Kovaleva V Raikhel NV Osborn RW Kester A Rees SB Torrekens S Vanleuven F Vanderleyden J Small cysteine-rich antifungal proteins from radish – their role in host-defense Plant Cell 1995 7 573 588 7780308 10.1105/tpc.7.5.573 PMC160805

[B6] Balandín M Royo J Gómez E Muniz LM Molina A Hueros G A protective role for the embryo surrounding region of the maize endosperm, as evidenced by the characterisation of ZmESR-6, a defensin gene specifically expressed in this region Plant Mol Biol 2005 58 269 282 10.1007/s11103-005-3479-1 16027978

[B7] Lay FT Schirra HJ Scanlon MJ Anderson MA Craik DJ The three-dimensional solution structure of NaD1, a new floral defensin from *Nicotiana alata* and its application to a homology model of the crop defense protein alfAFP J Mol Biol 2003 325 175 188 10.1016/S0022-2836(02)01103-8 12473460

[B8] Gaspar Y McKenna JA McGinness BS Hinch J Poon S Connelly AA Anderson MA Heath RL Field resistance to *Fusarium oxysporum* and *Verticillium dahliae* in transgenic cotton expressing the plant defensin NaD1 J Exp Bot 2014 doi:10.1093/jxb/eru021 10.1093/jxb/eru021 PMC3967090 24502957

[B9] Berg R Evaluation of spectral imaging for plant cell analysis J Microsc 2004 214 174 181 10.1111/j.0022-2720.2004.01347.x 15102064

[B10] Greenbaum L Schwartz D Malik Z Spectrally resolved microscopy of GFP trafficking J Histochem Cytochem 2002 50 1205 1212 10.1177/002215540205000907 12185198

[B11] Nielsen KJ Hill JM Anderson MA Craik DJ Synthesis and structure determination by NMR of a putative vacuolar targeting peptide and model of a proteinase inhibitor from *Nicotiana alata* Biochemistry 1996 35 369 378 10.1021/bi952228i 8555206

[B12] Wishart DS Bigam CG Holm A Hodges RS Sykes BD 1H, 13C and 15 N random coil NMR chemical shifts of the common amino acids. I. Investigations of nearest-neighbor effects J Biomol NMR 1995 5 67 81 10.1007/BF00227471 7881273

[B13] De Coninck B Cammue BPA Thevissen K Modes of antifungal action and *in planta* functions of plant defensins and defensin-like peptides Fungal Biol 2013 26 109 120 10.1016/j.fbr.2012.10.002

[B14] Seale JW Vordtriede PB Amino acid sequence variant alfalfa antifungal protein and its use in plant disease control US Pat US8067542

[B15] Ghag SB Shekhawat UKS Ganapathi TR Petunia floral defensins with unique prodomains as novel candidates for development of fusarium wilt resistance in transgenic banana plants PLoS One 2012 7 e39557 10.1371/journal.pone.0039557 22745785 PMC3382125

[B16] Ghag SB Shekhawat UKS Ganapathi TR Expression of C-terminal prodomain truncated Petunia floral defensins inhibit the growth of transgenic banana plants Curr Trends Biotechnol Pharm 2013 7 505 510

[B17] Janssen BJ Schirra HJ Lay FT Anderson MA Craik DJ Structure of *Petunia hybrida* defensin 1, a novel plant defensin with five disulfide bonds Biochemistry 2003 42 8214 8222 10.1021/bi034379o 12846570

[B18] Lay FT Mills GD Poon IK Cowieson NP Kirby N Baxter AA van der Weerden NL Dogovski C Perugini MA Anderson MA Dimerization of plant defensin NaD1 enhances its antifungal activity J Biol Chem 2012 287 19961 19972 10.1074/jbc.M111.331009 22511788 PMC3370180

[B19] van der Weerden NL Bleackley MR Anderson MA Properties and mechanisms of action of naturally occurring antifungal peptides Cell Mol Life Sci 2013 70 3545 3570 10.1007/s00018-013-1260-1 23381653 PMC11114075

[B20] van der Weerden NL Hancock RE Anderson MA Permeabilization of fungal hyphae by the plant defensin NaD1 occurs through a cell wall-dependent process J Biol Chem 2010 285 37513 37520 10.1074/jbc.M110.134882 20861017 PMC2988356

[B21] van der Weerden NL Lay FT Anderson MA The plant defensin, NaD1, enters the cytoplasm of *Fusarium oxysporum* hyphae J Biol Chem 2008 283 14445 14452 10.1074/jbc.M709867200 18339623

[B22] Lobo DS Pereira IB Fragel-Madeira L Medeiros LN Cabral LM Faria J Bellio M Campos RC Linden R Kurtenbach E Antifungal *Pisum sativum* defensin 1 interacts with *Neurospora crassa* cyclin F related to the cell cycle Biochemistry 2007 46 987 996 10.1021/bi061441j 17240982

[B23] Ramamoorthy V Cahoon EB Li J Thokala M Minto RE Shah DM Glucosylceramide synthase is essential for alfalfa defensin-mediated growth inhibition but not for pathogenicity of *Fusarium graminearum* Mol Microbiol 2007 66 771 786 10.1111/j.1365-2958.2007.05955.x 17908205

[B24] Thevissen K Cammue BPA Lemaire K Winderickx J Dickson RC Lester RL Ferket KKA Van Even F Parret AHA Broekaert WF A gene encoding a sphingolipid biosynthesis enzyme determines the sensitivity of Saccharomyces cerevisiae to an antifungal plant defensin from dahlia (*Dahlia merckii*) Proc Natl Acad Sci USA 2000 97 9531 9536 10.1073/pnas.160077797 10931938 PMC16899

[B25] Thevissen K Tavares PD Xu DM Blankenship J Vandenbosch D Idkowiak-Baldys J Govaert G Bink A Rozental S de Groot PWJ The plant defensin RsAFP2 induces cell wall stress, septin mislocalization and accumulation of ceramides in *Candida albicans* Mol Microbiol 2012 84 166 180 10.1111/j.1365-2958.2012.08017.x 22384976 PMC3405362

[B26] Thevissen K Warnecke DC Francois EJA Leipelt M Heinz E Ott C Zahringer U Thomma B Ferkel KKA Cammue BPA Defensins from insects and plants interact with fungal glucosylceramides J Biol Chem 2004 279 3900 3905 14604982 10.1074/jbc.M311165200

[B27] Amien S Kliwer I Márton ML Debener T Geiger D Becker D Dresselhaus T Defensin-like ZmES4 mediates pollen tube burst in maize via opening of the potassium channel KZM1 PLoS Biol 2010 8 1545 7885 10.1371/journal.pbio.1000388 PMC2879413 20532241

[B28] Spelbrink RG Dilmac N Allen A Smith TJ Shah DM Hockerman GH Differential antifungal and calcium channel-blocking activity among structurally related plant defensins Plant Physiol 2004 135 2055 2067 10.1104/pp.104.040873 15299136 PMC520777

[B29] Bednarek SY Wilkins TA Dombrowski JE Raikhel NV A carboxyl-terminal propeptide is necessary for proper sorting of barley lectin to vacuoles of tobacco Plant Cell 1990 2 1145 1155 2152159 10.1105/tpc.2.12.1145 PMC159962

[B30] Neuhaus JM Sticher L Meins F Boller T A short C-terminal sequence is necessary and sufficient for the targeting of chitinases to the plant vacuole Proc Natl Acad Sci USA 1991 88 10362 10366 10.1073/pnas.88.22.10362 1946457 PMC52928

[B31] Dombrowski JE Schroeder MR Bednarek SY Raikhel NV Determination of the functional elements within the vacuolar targeting signal of barley lectin Plant Cell 1993 5 587 596 8518558 10.1105/tpc.5.5.587 PMC160296

[B32] Frigerio L Foresti O Felipe DH Neuhaus J-M Vitale A The C-terminal tetrapeptide of phaseolin is sufficient to target green fluorescent protein to the vacuole J Plant Physiol 2001 158 499 503 10.1078/0176-1617-00362

[B33] Frigerio L Jolliffe NA Di Cola A Felipe DH Paris N Neuhaus J-M Lord JM Ceriotti A Roberts LM The internal propeptide of the ricin precursor carries a sequence-specific determinant for vacuolar sorting Plant Physiol 2001 126 167 175 10.1104/pp.126.1.167 11351080 PMC102291

[B34] Saalbach G Rosso M Schumann U The vacuolar targeting signal of the 2S albumin from Brazil nut resides at the C-terminus and involves the C-terminal propeptide as an essential element Plant Physiol 1996 112 975 985 10.1104/pp.112.3.975 8938406 PMC158024

[B35] Wilkins TA Raikhel NV Expression of rice lectin is governed by two temporally and spatially regulated mRNAs in developing embryos Plant Cell 1989 1 541 549 2535550 10.1105/tpc.1.5.541 PMC159788

[B36] Bol JF Linthorst HJM Cornelissen BJC Plant pathogenesis-related proteins induced by virus-infection Annu Rev Phytopathol 1990 28 113 138 10.1146/annurev.py.28.090190.000553

[B37] Tabe L Wardley-Richardson T Ceriotti A Aryan A McNabb W Moore A Higgins T A biotechnological approach to improving the nutritive value of alfalfa J Anim Sci 1995 73 2752 2759 8582868 10.2527/1995.7392752x

[B38] Bevan M Binary *Agrobacterium* vectors for plant transformation Nucleic Acids Res 1984 12 8711 8721 10.1093/nar/12.22.8711 6095209 PMC320409

[B39] Yi CX Zhang J Chan KM Liu XK Hong Y Quantitative real-time PCR assay to detect transgene copy number in cotton *Gossypium hirsutum* Anal Biochem 2008 375 150 152 10.1016/j.ab.2007.11.022 18078801

[B40] Davis SJ Vierstra RD Soluble, highly fluorescent variants of green fluorescent protein (GFP) for use in higher plants Plant Mol Biol 1998 36 521 528 10.1023/A:1005991617182 9484447

[B41] Conlan BF Gillon AD Barbeta BL Anderson MA Subcellular targeting and biosynthesis of cyclotides in plant cells Am J Bot 2011 98 2018 2026 10.3732/ajb.1100382 22081413

[B42] Schnölzer M Alewood P Jones A Alewood D Kent SBH In situ neutralization in Boc-chemistry solid phase peptide synthesis Int J Pept Protein Res 1992 40 180 193 1478777 10.1111/j.1399-3011.1992.tb00291.x

